# Primary hepatic angiosarcoma: a systematic review

**DOI:** 10.1097/MS9.0000000000001584

**Published:** 2024-01-04

**Authors:** Santiago Rojas, Carlos Eduardo Rey Chaves, Sofía Robledo, Danny Conde, Juan Carlos Sabogal Olarte

**Affiliations:** a Estudiante de posgrado Cirugía General; b Estudiante de pregrado, Pontificia Universidad Javeriana, Facultad de Medicina; c Cirugia Hepatobiliar y pancreática, Hospital Universitario Mayor Méderi, Universidad el Rosario; d Cirugía Hepatobiliar y páncreatica, Pontificia Universidad Javeriana, Facultad de Medicina, Hospital Universitario San Ignacio, Bogotá, Colombia

**Keywords:** liver angiosarcoma, liver tumour, outcomes, surgery, treatment

## Abstract

**Background:**

Hepatic angiosarcoma is a rare type of tumour. In adults, the diagnosis remains challenging as the clinical manifestations are generally nonspecific and are usually made too late when complications or metastases are already present, associated with a poor prognosis. Due to the lack of agreement regarding the optimal treatment approach, a comprehensive analysis of the evidence in the literature on the surgical and survival outcomes in terms of disease-free survival time (DFS) and overall survival (OS) for patients treated for primary hepatic angiosarcoma is needed.

**Study design:**

A systematic review of articles published in Pubmed, EMBASE, and Scopus, from 2000 to 2023 with the search terms hepatic angiosarcoma and liver resection or hepatectomy. Pooled individual data concerning the prognosis following various therapies was analyzed.

**Results:**

A total of 15 articles involving 886 patients were eligible for inclusion. The male population represents 66.2% (*n*=586) of the population, with a median age of 57 years (41–66). The median survival rate was 7 months. The median OS after surgical resection was 17.18 months (SD 12.6) vs. 3.72 months (SD 4.6) for patients treated without a surgical approach. The median DFS was 15.2 months (SD 11).

**Conclusion:**

Primary liver angiosarcoma remains a surgical challenge with a poor prognosis even with complete surgical resection and adjuvant therapy. Surgical management shows increased overall survival in comparison with non-surgical management. Early diagnosis could change the natural history of the disease. The literature available is scarce, and further studies are required to create standardized diagnostic and treatment protocols.

## Background

Highlights

- Hepatic angiosarcoma remains to be a surgical challenge.
- Better oncologic outcomes are evidenced in patients treated with a surgical approach
- We require surgical clinical practice guidelines in order to standardize the treatment protocols of liver angiosarcoma.

Hepatic angiosarcoma is a high-grade vascular neoplasm that occurs predominantly in individuals over 60 years of age, with a male-to-female ratio of 3:1. Although it represents 1–2% of liver tumours, it is the third most common primary hepatic tumour. The exact causal agent has not been clarified, but its occurrence is associated with exposure to environmental toxins such as arsenic, vinyl chloride, radiation, thorium dioxide (thorotrast), exogenous estrogens, and anabolic steroids. In 75% of the cases, the exact aetiology remains undetermined^1–3^.

The diagnosis of liver angiosarcoma remains challenging as the clinical manifestations are generally nonspecific, and in most cases, there are already complications or metastases at the time of diagnosis. Imaging studies can aid in diagnosis; however, in many cases, it is not possible to differentiate it from other liver tumours that also exhibit hypervascularization, such as hemangiomas, hemangioendothelioma, or carcinomas^4,5^. Computed tomography is considered the gold standard imaging modality. Findings can include infiltrative lesions, multiple nodules, or dominant solitary masses. A definitive diagnosis is made through histopathological findings obtained from a biopsy. Laparoscopic or open biopsy is preferred over trans jugular or image-guided percutaneous biopsy, as the latter may yield nonspecific or inconclusive results or may be associated with tumour haemorrhage within 48 h post-procedure^6,7^.

Currently, there are no standardized guidelines for the management of patients with hepatic angiosarcoma. This is due to the rarity of the condition and its poor prognosis, with a median survival of 6 months without treatment^8^. Furthermore, in many cases, patients present with acute lethal conditions such as liver failure, massive haemorrhage, or tumour rupture^1^. While there is limited literature on management, a consistent finding is that complete or radical surgical resection is the most effective treatment for non-metastatic solitary lesions^1–3^.

Zheng and colleagues conducted a meta-analysis of 64 cases reported between 2002 and 2012 in Asia and Europe, which demonstrated that the median overall survival (OS) of all patients was 5 months, whereas those who underwent local resection with or without chemotherapy (QT) had a survival of ~17 months. Sixty-six percent of patients who received this management survived beyond 6 months^9^. Ideally, surgical resection should be performed in all patients with resectable disease; however, this is only achieved in 20% of patients since most are diagnosed with multiple nodular tumours spread across both hepatic lobes and metastatic disease. Some authors even proposed liver transplantation (LT) as a treatment option for patients presenting with liver failure, bilobar masses, and hepatic rupture. Nonetheless, the results were dismal, with recurrence diagnosed after 5 months, 77.2% of the patients dying of tumour recurrence, and a median OS of 7.2 months^10^. This supports the fact that more than seeking a radical resection, early detection of angiosarcoma is the main factor associated with improved survival. Tripke *et al*.^11^ report an OS of 59 months for patients with early diagnosis and R0 resection.

Literature has reported case reports, series, and pooled analyses of liver sarcomas, but none of them have focused on angiosarcoma, as it is the one with the worst prognosis and fewer therapeutic options available due to its aggressive behaviour. The present systematic review aimed to study the evidence in the literature on the surgical and survival outcomes in terms of disease-free survival time (DFS) and OS for patients treated with liver resections for primary hepatic angiosarcoma to provide further useful evidence for decision-making in clinical practice.

## Methods

This systematic review was reported according to the Preferred Reporting Items for Systematic Review and Meta-Analysis (PRISMA) Statement, Supplemental Digital Content 1, http://links.lww.com/MS9/A342, as well this work has been reported in line with AMSTAR (Assessing the methodological quality of systematic reviews), Supplemental Digital Content 2, http://links.lww.com/MS9/A343. Ethical approval and informed consent were not needed for this paper.

### Criteria for considering studies

The study inclusion and exclusion criteria included original, peer-reviewed retrospective or prospective studies involving humans that report surgical outcomes (morbidity, 30-day mortality rate) and survival outcomes (DFS and OS) of patients with liver resection with primary angiosarcoma, and/or oncologic management such as chemotherapy, radiotherapy, trans-arterial chemoembolization, among others. Letters, commentaries, conference abstracts, reviews, and meta-analyses were excluded. Also, papers without information based on the outcomes or data that could not be analyzed were excluded.

### Search strategies and study selection

Two independent reviewers performed systematic research of the literature from January 2000 to April 2023 within Pubmed, EMBASE, and Scopus databases with the following keywords: hepatic angiosarcoma and liver resection or hepatectomy. The details of the search strategies can be found in Appendix, Supplemental Digital Content 3, http://links.lww.com/MS9/A344. After excluding duplicate studies, two independent reviewers (S.R. and C.R.) screened the titles and abstracts for potentially eligible studies. Then, full texts were retrieved to identify the potentially eligible studies that met the inclusion and exclusion criteria. Any differences in study selection were solved by consensus among all authors. Additional articles were retrieved through a manual search of the bibliographies of the selected articles.

### Outcomes

The primary outcomes included the OS and DFS. The secondary outcomes were morbidity and mortality and the ability to describe alternative treatment strategies for patients with primary hepatic angiosarcoma.

### Data collection

Two authors independently extracted the data from the included studies, which included study design, publication year, country, clinical characteristics, treatment strategy, OS at 1, 3, and 5 years, DFS, morbidity, and mortality, among others.

### Study quality assessment

We used the Newcastle–Ottawa quality assessment scale for systematic reviews to evaluate the risk of bias and applicability of the included studies by two different evaluators, and then matched and compared the results of the quality assessment.

#### Data analysis

Descriptive statistics of all study parameters were provided according to the nature of the variable. The distribution of the variables was assessed according to the Kurtosis/Skewness test. Continuous variables were summarized by means, or medians, and standard deviation, or interquartile ranges, according to their nature and distribution. Categorical data were summarized by frequency and proportion. Data analysis was performed using STATA 17.

## Results

### Study selection

**Figure 1:**
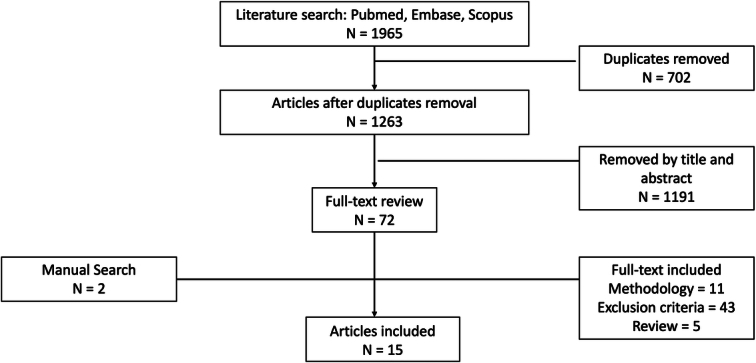
Preferred Reporting Items for Systematic Review and Meta-Analysis (PRISMA) flow chart of the eligible study selection.

A total of 1965 references were obtained from PUBMED, EMBASE, and SCOPUS. Duplicates were removed, and 1263 articles were eligible for title and abstract review. Finally, 72 articles were included and evaluated for a full-text analysis. After applying selection criteria and manually adding two articles, 15 studies were included in the systematic review^10,12–26^. All the included literature has a retrospective cohort design with no intervention. A flow chart summarizing the research process is displayed in Figure 1.

### Methodological quality appraisal

All the included studies for the final analysis have a retrospective design. Newcastle–Ottawa quality assessment^24^ tools were applied, and all the studies have acceptable quality and a low risk of bias. Of the studies included, 37.5% were conducted in the Asian population, 37.5% in the United States of America, and 25% in the European population.

### Patient and tumour characteristics

A total of 886 patients were included in our synthesized population. From the reported data, the white race corresponds to most of the population (70%, *n*=621). The median age of the population analyzed was 57 years (Range 41–66). Men constituted 66.2% (*n*=586) of the population, and female patients 33.7% (*n*=300). With the available data, 0.56% of the patients had a prior infection with the Hepatitis B virus, and 0.11% had exposure to carcinogens such as polyvinyl chloride (PVC) or thorotrast. Nevertheless, most of the studies do not report associated risk factors. Only 0.9% of the entire population initially presented with a liver rupture. At the time of diagnosis, 28.32% (*n*=251) of the patients presented with metastatic disease, with bone, lung, spleen, and peritoneum as the primary sites involved. Patients presented with multiple liver tumours in 10.60% (*n*=94), and the mean size of the tumours was 11.18 cm (SD 5.84) (See Supplemental Digital Content 4 Table S1, http://links.lww.com/MS9/A372).

### Treatments

Surgical resection plus adjuvant chemotherapy was indicated for most of the population (41.64%, *n*=369). Surgical resection alone was performed in 22.23% (*n*=197) of the patients; palliative chemotherapy was preferred for 32.73% (*n*=290); trans-arterial chemoembolization (TACE) was used in 1.05% (*n*=9); and radiotherapy in 2.37% (*n*=21) (See Supplemental Digital Content 4 Table S1, http://links.lww.com/MS9/A372).

### Overall survival and disease-free survival

Of the data analyzed, 86.6% of the studies reported OS at 1 year, 66.66% at 3 years, and 5. With the collected data, the median OS at 1 year was 30% (range 5.26–100%). At 3 years, the median OS was 4.05% (Range 0–80%). And at 5 years, the median OS was 0% (0–40%). The median survival time was 7 months (IQR: 2–59 months). Four studies report a disease-free survival time with a mean time to illness relapse was 15.2 months (SD 11.7).

OS was compared between patients who underwent surgical management versus patients who did not receive this approach. The median OS for patients who underwent surgical management was 17.1 months (SD 12.6) vs. 3.7 months (SD 3.6) for the patients who did not (See Supplemental Digital Content 4 Table S1, http://links.lww.com/MS9/A372).

## Discussion

Hepatic angiosarcoma is an aggressive, rare tumour, accounting for just 2% of primary hepatic malignancies, but among all of them, it has the worst prognosis^8^. It is composed of undifferentiated spindle or pleomorphic cells within a myxoid matrix that grow into the lumen of vascular sinusoidal spaces in the liver, causing liver atrophy. The main factors related to poor prognosis are advanced disease at the time of diagnosis, spontaneous hepatic bleeding, rupture, and liver failure^10,12–18^. In this study, we found that approximately 28.32% of the patients presented with metastasis at the time of presentation, and less than 1% presented liver rupture. Also, less than 1% of the patients had prior infection with the Hepatitis B virus and exposure to carcinogens such as PVC or thorotrast, in contrast with the previously reported 25% exposure in a nationwide survey for patients with this tumour between 1964 and 1974 in the USA^18–21^. Therefore, literature is scarce regarding possible associated carcinogens related to liver angiosarcoma.

Currently, there is no consensus regarding the most appropriate therapy for this aggressive tumour. Options vary from anatomical, partial, liver resections or liver transplant, other non-surgical options such as radiotherapy, systemic chemotherapy, and transcatheter arterial chemoembolization (TACE) are as well described, however there are no guidelines or standardized treatments, but survival benefits have been shown in patients with surgical radical excision with or without adjuvant chemotherapy^21–23^. Radiation therapy usually does not work as this tumour is radioresistant and there is no standardized chemotherapy regimen. In most case series and reports, chemotherapy was used for palliative rather than curative purposes. Various regimens have been proposed in the literature, such as doxorubicin as a single agent and weekly paclitaxel, gemcitabine, and doxorubicin-based schemes, which deserve further studies and consideration^24–29^. Recently, bevacizumab, sorafenib, and pazopanib have been considered as possible targeted drugs^12^. Immunotherapy targeting the programmed death 1 (PD-1) receptor and its ligand (PD-L1) is also being studied for sarcomas, as are tyrosine kinase inhibitors due to their broad spectrum of inhibitory action on tumour angiogenesis and growth^30,31^.

Previously, it was reported that less than 20% of the patients had resectable disease. In our review, only 10% of the patients presented with multiple masses, and ~30% were Stage IV, contrasting with this fact. Based on this systematic review, surgical resection of angiosarcoma, whenever it’s feasible, is the best treatment option available, despite early recurrence rates. The population analyzed showed a significant difference in survival for those who underwent surgical resection (Surgery 17.1 vs. Non surgery 3.7 months), probably reflecting an earlier stage, a less comorbid population, and the benefit of surgical resection as seen in other sarcomas. Zheng’s meta-analysis also showed that survival was extended to up to 84 months when surgery was combined with adjuvant chemotherapy for solitary or confined hepatic tumours. 23 out of 30 (76.7%) patients survived more than 6 months when they were able to undergo liver resection alone or in combination with adjuvant therapy^9^. Despite this, high tumour recurrence has been reported with a mean DFS of 15.2 months (SD 11.7) even when there was R0 resection and no evidence of metastatic disease.

LT has evolved as a viable treatment choice for liver neoplasms displaying favourable characteristics, such as hepatocellular carcinoma, without any signs of metastatic spread^20^. However, the results of hepatic angiosarcoma have been disastrous. In our analysis, we included the largest retrospective study, which included 22 patients reported to the European Liver Transplant Registry who were transplanted for angiosarcoma. They had a median survival of 6 months, with recurrence diagnosed within 5 months after LT, and 17 (77%) of the patients died of tumour recurrence. All patients died before the end of the second post transplantation year; the other five of early infections. The longest survival was 23 months. LT was performed in cases of a painful multinodular tumour (68%), liver failure of unclear aetiology (23%), one patient with a putative diagnosis of hepatocellular carcinoma, and one with hemangioendothelioma. Surgical complications occurred in 28% of the patients, and infectious complications occurred in 55% of them. Therefore, they concluded that the differential diagnosis of vascular liver tumours must be refined before considering LT, and if it is not clear, a large tissue biopsy should be obtained with at least a 6-month observance period on the waiting list to evaluate disease progression. And second, and most important for our scope, is that hemangiosarcoma is an absolute contraindication to LT^10^.

Other options include TACE, which is classically described as an emergency measure in cases of haemorrhage secondary to tumour rupture with palliative intention and does not significantly affect tumour size or patient survival. Some authors have proposed it as a therapeutic measure in metastatic and unresectable angiosarcoma; however, there is insufficient evidence to support that^15^.

Among the limitations of our study were the limited literature available for analysis and the heterogeneity of the population. All the studies are retrospective, and some of them lack detailed information about chemotherapy regimens, TACE, liver transplantation, and comorbidities that can impact clinical outcomes and therapeutic interventions, and those limitations reflect the requirement of standardized reports in order to understand this pathology. Our study increases the evidence regarding primary angiosarcoma of the liver, demonstrating a poor prognosis even with complete surgical resection associated with adjuvant therapy.

## Conclusion

Primary liver angiosarcoma remains to be a surgical challenge with a poor prognosis even with complete surgical resection and adjuvant chemotherapy or radiotherapy. Nevertheless, in a high proportion of cases, at the time of diagnosis, metastatic disease is already evident, and there are scarce therapeutic regimen studies. In general, the outcome for patients who received aggressive treatment was more favourable compared to those who underwent conservative treatment, especially surgical resection, prolonging survival significantly. Often, patients who opt for conservative treatment find it challenging to pursue other therapies due to the rapid progression of their illness and/or poor functional state with comorbidities. Then, early diagnosis should be the essential goal, as further studies are required to create standardized diagnostic and treatment protocols. Given the tumour’s aggressiveness and unfavourable prognosis, collaborative endeavours across multiple centres are essential. Creating a global database of patients with this rare disease should help identify the best clinical approaches and effective therapeutic approaches.

## Ethical approval

All procedures performed in studies involving human participants were in accordance with the ethical standards of the institutional and/or national research committee, and with the 1964 Helsinki Declaration and its later amendments or comparable ethical standards.

## Consent

Does not apply.

## Sources of funding

This research did not receive any specific grant from funding agencies in the public, commercial, or not-for-profit sectors.

## Author contribution

S.R. and C.E.R.C. had the research idea. S.R., S.R., and C.E.R.C. participated in drafting the article and revised it critically for important intellectual content. S.R., S.R., C.E.R.C., D.C., and J.S. made substantial contributions to the conception and design, acquisition of data, analysis, and interpretation of data.

## Conflicts of interest disclosure

None of the authors declare any conflicts of interest.

## Research registration unique identifying number (UIN)

Registration in process in PROSPERO ID 476052 “Review Ongoing Not Yet Published”.

## Guarantor

Carlos Eduardo Rey Chaves.

## Data availability statement

The datasets used and/or analyzed during the current study are available from the corresponding author upon reasonable request.

## Provenance and peer review

Not commissioned, externally peer-reviewed.

## Supplementary Material

SUPPLEMENTARY MATERIAL
