# Supplementary material for: Primary hepatic angiosarcoma: a systematic review
Source: Ann Med Surg (Lond). 2024 Jan 4;86(3):1601–5. doi: 10.1097/MS9.0000000000001584 (PMC10923304; doi:10.1097/MS9.0000000000001584)
Supplement: SUPPLEMENTARY MATERIAL [file ms9-86-1601-s003.docx]

**Supplementary material**

*“Hepatic angiosarcoma or angiosarcoma” AND “overall survival OR surgical outcomes”, AND “hepatectomy OR liver resection”*

*“Angiosarcoma AND hepatectomy”, “liver AND cancer AND angiosarcoma” “Hepatic AND Angiosarcoma AND hepatectomy”, “liver resection”, “hepatectomy”.*

| **NewCastle Ottawa Score** |
| --- |
| Molina et al (14): Good Quality (3/1/3)  Weitz et al.(8): Fair Quality (2/2/2)  Kim et al (13): Fair Quality (2/2/2)  Matthaei et al (19): Fair Quality (2/2/1)  Zhou et al (21): Fair Quality (2/1/3)  Duan y Li (17): Fair Quality (2/1/2)  Orlando et al (16): Good Quality (3/1/3)  Hur et al. (12) : Fair Quality (2/1/3)  Lin et al. (23): Fair Quality (2/1/2)  Huang et al (22): Good Quality (3/2/3)  Wilson et al (15): Fair Quality (2/2/2)  Tripke et al (10): Good Quality (3/1/3)  Martinez et al. (11): Fair Quality (2/1/3)  Mangla et al (18): Good Quality (3/1/3)  Liao et al (20): Good Quality (3/1/3) |
